# Supplementary material for: A Randomized Controlled Crossover Lifestyle Intervention to Improve Metabolic and Mental Health in Female Healthcare Night-Shift Workers
Source: Nutrients. 2025 Oct 24;17(21):3342. doi: 10.3390/nu17213342 (PMC12608408; doi:10.3390/nu17213342)
Supplement: Supplementary file 1 [file nutrients-17-03342-s001.zip › nutrients-3911177-supplementary.pdf]

Article

A Randomized Controlled Crossover Lifestyle Intervention to Improve Metabolic and Mental Health in Female Healthcare Night-Shift Workers

Laura A. Robinson [1,2,*], Sarah Lennon [1,2], Alexandrea R. Pegel [2], Kelly P. Strickland [2,3], Christine A. Feeley [2], Sarah O. Watts [2], William J. Van Der Pol [4], Michael D. Roberts [5], Michael W. Greene [1] and Andrew D. Frugé [2,5]

Supplementary Materials:

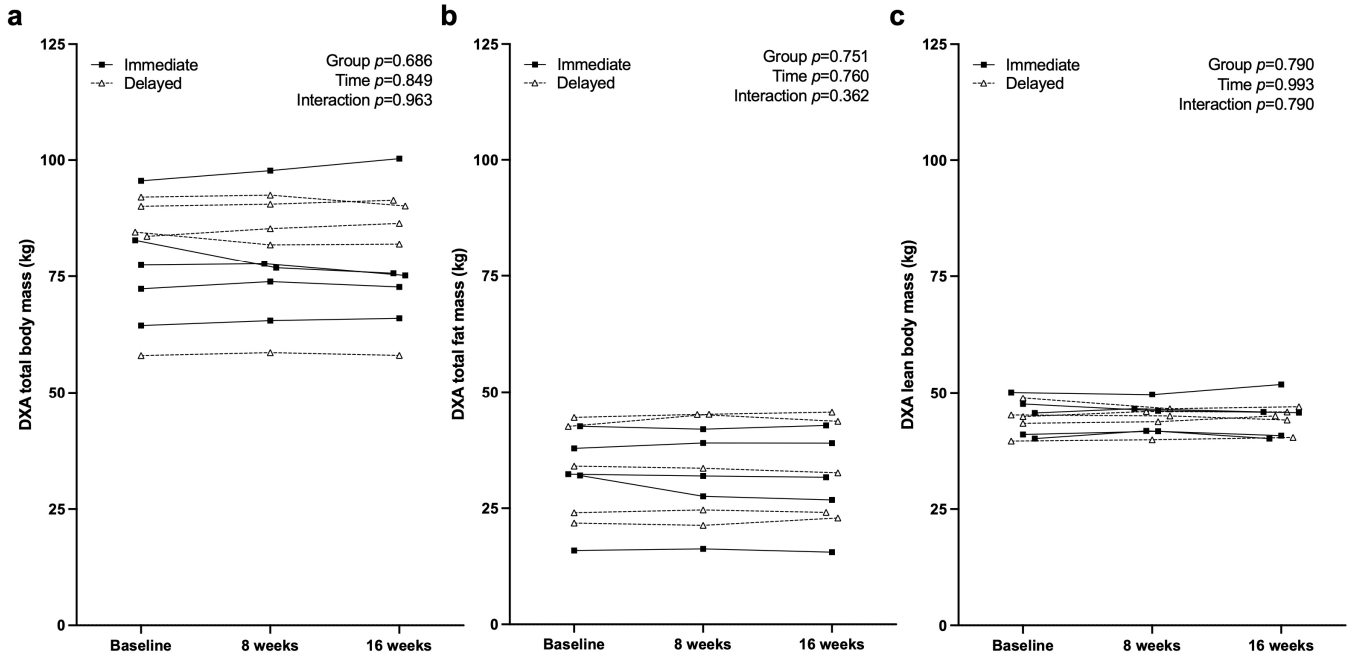

Supplemental Figure S1. Individual changes in DEXA **(a)** total body mass (kg), **(b)** total fat mass (kg), and **(c)** lean body mass (kg), by timepoint.

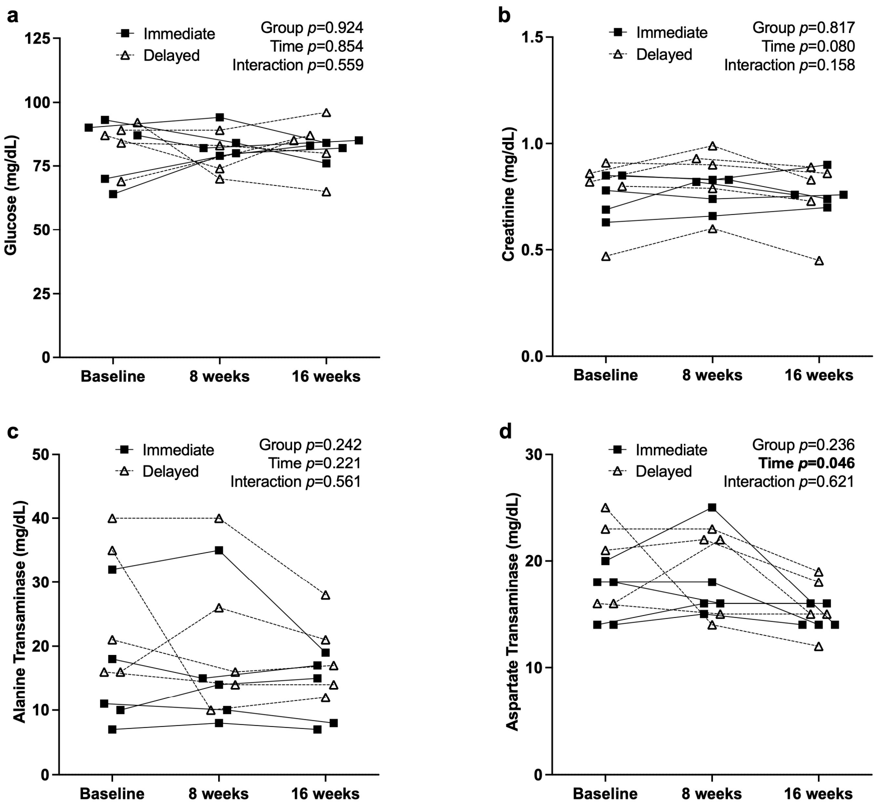

Supplemental Figure S2. Individual changes in **(a)** Glucose (mg/dL), **(b)** Creatine (mg/dL), **(c)** Alanine Transaminase (mg/dL), **(d)** Aspartate Transaminase (mg/dL), by timepoint.

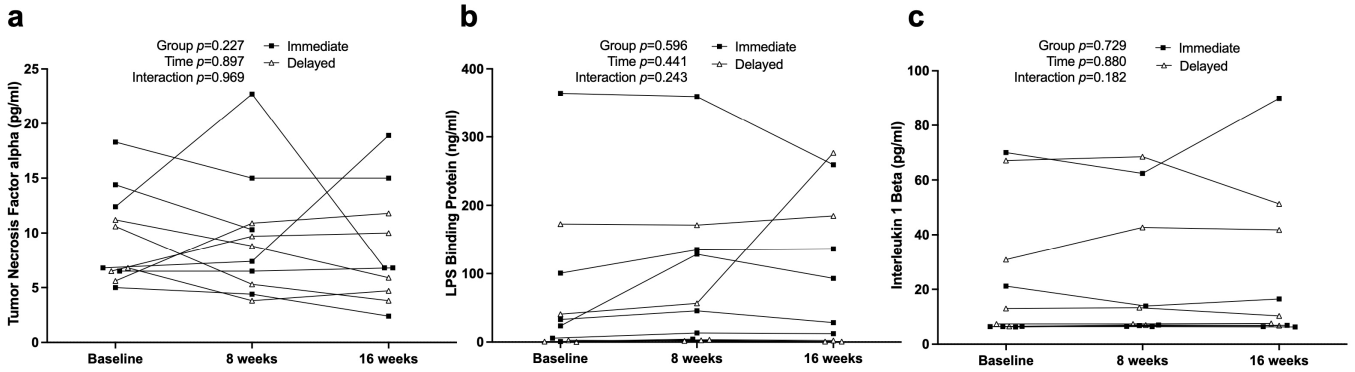

Supplemental Figure S3. Individual changes in **(a)** Tumor Necrosis Factor alpha (pg/ml), **(b)** LPS Binding Protien (ng/ml), **(c)** Interleukin 1 Beta (pg/ml), by timepoint.
